# The role of protective genetic variants in modulating epigenetic aging

**DOI:** 10.1007/s11357-025-01548-2

**Published:** 2025-02-10

**Authors:** Yosra Bejaoui, Luma Srour, Abeer Qannan, Junko Oshima, Chadi Saad, Steve Horvath, Hamdi Mbarek, Nady El Hajj

**Affiliations:** 1 College of Health and Life Sciences, Hamad Bin Khalifa University, Qatar Foundation, Doha, Qatar; 2 Department of Laboratory Medicine and Pathology, University of Washington, Seattle, WA 98105 USA; 3 Department of Clinical Cell Biology and Medicine, Graduate School of Medicine, Chiba University, Chiba, Japan; 4 Qatar Genome Program, Qatar Precision Health Institute, Qatar Foundation, Doha, Qatar; 5 Altos Labs, San Diego, USA; 6 Department of Human Genetics, David Geffen School of Medicine, University of California Los Angeles, Los Angeles, CA 90095 USA; 7 College of Science and Engineering, Hamad Bin Khalifa University, Qatar Foundation, Doha, Qatar

**Keywords:** Unimodal antigeroid syndromes, Epigenetic aging, Protective variants, APOE, PCSK9, Progeroid syndromes

## Abstract

Several progeroid syndromes’ causative mutations have been linked to epigenetic age acceleration as measured via several epigenetic clocks. At the same time, several protective variants have also been discovered that can reduce the risk of developing certain age-related disorders. However, the impact of these protective variants on epigenetic aging has not been well elucidated. Our research, which involved screening over 14,669 healthy individuals enrolled in the Qatar BioBank (QBB) and sequenced by the Qatar Genome Project (QGP), identified individuals carrying protective variants against age-related disorders, including Alzheimer’s disease (AD), type 2 diabetes (T2D), and atherosclerosis. In this study, we measured methylation levels in blood DNA using the EPIC v2 arrays. In addition, epigenetic age was calculated using various epigenetic clocks. Our analysis revealed that the APOE*E2 protective variant reduces the rate of GrimAge epigenetic aging when compared to individuals with the APOE4 AD risk allele. Furthermore, our differential DNA methylation analysis discovered the association of the *PCSK9* protective variant with specific biological processes related to immune function and the cardiovascular system. In conclusion, APOE*E2 protective variants have a positive impact on epigenetic aging, while *PCSK9* protective variants have a significant effect on DNA methylation signatures. Further studies are needed to better understand the underlying mechanisms by which protective variants influence epigenetic aging, particularly the role of APOE*E2 protective variants in biological aging. Furthermore, additional research is required to fully uncover the processes that might enable specific targeted therapies to mimic the effects of beneficial mutations, such as LOF variants in *PCSK9*, in reducing the risk of geriatric disorders.

**Supplementary Information:**

The online version contains supplementary material available at 10.1007/s11357-025-01548-2.

## Introduction

Most aging studies have focused on the analysis of human subjects with long health spans and life spans, i.e., centenarians [1]. The analysis of individuals displaying resistance against specific diseases such as cardiovascular disease, dementias of Alzheimer’s type (DATs), and type 2 diabetes (T2D) has been less investigated. These particular cases are referred to as “unimodal antigeroid syndromes” describing individuals carrying variants that provide enhanced protection against specific aging-related diseases [2]. The notion of “Antigeroid Syndromes” was first introduced by George Martin, who encouraged researchers to study how beneficial variants might affect aging. In his view, it would be important that geriatric research not only focuses on harmful variants in progeroid syndromes but also on protective variants that confer resistance/resilience for certain age-related disorders.

One of the well-studied genes in this context is the *APOE* gene, the major susceptibility gene for late-onset Alzheimer’s disease (AD), with three common alleles: *APOE2*, *E3*, and *E4*, which confer different levels of disease risk. The E3/E3 genotype is the most prevalent and has a neutral effect on AD risk [3]. However, carrying one or two *APOE4* alleles increases the risk of AD and lowers the age of onset [4]. Conversely, the *APOE2* allele is a protective allele that reduces the risk and delays the onset of AD. *APOE2*’s protective allele has a wide range of potential mechanisms that may include resistance to protein denaturation, more favorable protein–protein interactions, inhibition of amyloid β monomer transformation into toxic oligomers, enhanced degradation, and clearance of amyloid β deposits, protection of neurons from apoptosis, improved anti-inflammatory and antioxidant functions, reduction of neurofibrillary tangles, and support for synaptic integrity [5]. Another example of a unimodal antigeroid syndrome related to atherosclerosis comes from the discovery of low levels of LDL cholesterol associated with loss-of-function mutations at *PCSK9* (including haploinsufficiency) [6]. Monoclonal antibodies have been developed to decrease PCSK9 levels, which protect individuals from atherosclerosis and ischemic heart disease [7]. In addition to *PCSK9*, the *APOC3* null mutation R19* (rs76353203) profiled in the Amish population is associated with cardio-protective effects, including significantly higher levels of HDL-C and lower levels of triglycerides (TG) and total cholesterol levels in comparison to non-carriers [8]. Furthermore, the carriers of protein-truncating variants in *SLC30A8* have been reported to exhibit a 65% lower risk of T2D compared to non-carriers, indicating that the gene’s loss of function protects against diabetes [9]. Similarly, a mouse model that carries the protective p.Trp138* allele showed a 50% increase in insulin secretion in response to hyperglycemia [10]. These findings are supported by human studies demonstrating that particular loss-of-function missense mutations in *SLC30A8* enhance proinsulin processing and insulin responsiveness to glucose, conferring protection against T2D [11].

Studying allelic variants that provide robust resistance to age-related diseases can be fundamental in understanding aging mechanisms or specific physiological functions that deteriorate during aging. Therefore, we investigated epigenetic age acceleration (EAA) and differential DNA methylation in individuals carrying protective variants to assess whether the studied protective variants reduced the rate of EAA and conferred specific DNA methylation signatures.

## Methods

### Allele frequency Qatar Genome Project

All samples were sequenced on Illumina HiSeq X instruments (Illumina, San Diego, CA, USA) to a target average depth of coverage of 30 × . Quality control of the produced FastQ files was performed using FastQC (v0.11.2). The read mapping, variant calling, and joint variant calling were performed using Sentieon’s DNASeq (v201808.03) pipeline following the BWA-GATK Best Practice Workflow and using the GRCh38/hg38 reference genome [12]. The produced individual gVCF files were jointly called to produce one multisample VCF file (msVCF) for the whole cohort, allowing the allelic frequencies calculation.

### DNA methylation profiling

DNA methylation profiling was performed using the Infinium MethylationEPIC v2.0 BeadChip according to the manufacturer’s protocol. In summary, 500 ng DNA for each sample was bisulfite converted using the EZ DNA Methylation Kit (Zymo Research, Irvine, CA, USA). Following bisulfite conversion, the DNA was amplified, enzymatically fragmented, and hybridized to the Infinium MethylationEPIC BeadChips. The arrays were then scanned via the Illumina iScan. To avoid batch effect, all samples were processed simultaneously and randomized on the arrays. Raw intensity data (IDAT) files were exported and analyzed with the R software (version 4.2.2).

The IDAT files were analyzed using the RnBeads package [13, 14]. Quality control and preprocessing steps include (1) filtering out probes overlapping SNPs (*n* = 9981) and (2) filtering out probes and samples with the highest fraction of unreliable measurements using greedycut (*n* = 10,490). In total, 20,471 probes were removed, and all samples were retained. (3) Normalizing data was done using Dasen, followed by excluding probes located on sex chromosomes (*n* = 23,143). Overall, 875,028 probes were retained for further differential DNA methylation analysis. The relative proportion of white blood counts was estimated using the Houseman *et al.* method (2014). This method is based on blood-derived DNA methylation signatures measured via the Illumina HumanMethylationEPIC array that can estimate the proportions of neutrophil, monocyte, B-lymphocyte, natural killer, and CD4 + and CD8 + T-cell fractions [15]. Differential methylation analysis was conducted at both the CpG site and region level (promoters, CpG Islands, and 5 Kb Tiling windows). Cellular heterogeneity was accounted for using the Houseman method followed by limma-based analysis to adjust for age and gender as covariates.

Gene set analysis was conducted using the methylglm function as part of the methylGSA to identify enriched biological processes, molecular functions, and cellular components associated with differentially methylated CpG sites after adjusting for the number of CpG sites per gene [16]. eFORGE 2.0 was employed to identify the overlap of differentially methylated CpG sites with DNAse 1 hypersensitive sites (DHS) across multiple tissues, using data from the Roadmap Epigenomics project. eFORGE 2.0 for the EPIC v2 arrays is still under development; therefore, only common DMPs between EPIC v1 and v2 arrays were analyzed. The FDR method was used to correct *p-value*s for multiple testing.

### Calculating DNA methylation age and epigenetic age acceleration

Epigenetic age acceleration (EAA) was measured using several epigenetic clocks, Horvath [17], Hannum [18], SkinBlood [19], GrimAge [20], PhenoAge [21], and FitAge [22], in addition to  extrinsic epigenetic age acceleration (EEAA) [23] and intrinsic epigenetic age acceleration (IEAA) [23]. DNA methylation (DNAm) age was calculated using the web-based DNAm age calculator (https://dnamage.genetics.ucla.edu/). Epigenetic age acceleration “EAA” is the residual from a regression of estimated epigenetic age on chronological age. An individual’s age acceleration (positive score) or “age deceleration” (negative score) was defined according to the direction of the deviation.

EAA, by definition, is already adjusted for age. Other covariates previously shown to be associated with EAA, such as sex [17, 19, 21, 24, 25], were further adjusted for. Ethnicity adjustment was omitted since all individuals studied were Qatari [26]. If a covariate had already been incorporated during the training of the epigenetic clock, it was excluded from the specific model (e.g., sex and smoking in GrimAge).

Data from the publically available GEO dataset (GSE75310, GSE100825, GSE182991, GSE131752, GSE214297) of samples characterized as progeroid syndromes: 8 classical and seven non-classical Hutchinson Gilford progeria syndrome (HGPS) samples, 6 atypical and 20 classical Werner syndrome samples, 4 dyskeratosis congenita samples, and 7 samples from Berardinelli–Seip congenital lipodystrophy type 2 (CGL2) patients. In all those samples/datasets, DNA methylation profiling was performed on blood DNA. In addition, DNA methylation data from two patients suffering from cerebroretinal microangiopathy with calcifications and cysts were included in the progeroid group to compare with samples carrying protective variants. Supplementary Table 1 shows the progeroid samples with age > 20 years included for further analysis.

## Results

### Frequency of protective variants in the QGP cohort

We used whole genome sequencing data from > 14,669 participants from QGP, to identify individuals carrying the following protective genetic variants: *APOE2 ε2ε2*: rs429358 (T;T) + rs7412 (T;T); *PCSK9* c.2037C > A, (p.Cys679*) and c.426G > C, (p.Tyr142*); *APOC3* c.55C > T, (p.Arg19*); *SLC30A8* c.412C > T, (p.Arg138*); or *ANGPTL3* c.385G > T, (p.Glu129*) or c.50_51delinsGA (p.Ser17*). We identified 447 individuals harboring a protective variant against atherosclerosis, including 422 heterozygous individuals and 13 homozygous for the *APOC3* p.Arg19* variant (Table 1). Furthermore, 12 heterozygous individuals with the *PCSK9* p.Cys679* protective variant were identified. The minor allele frequency (MAF) for this LOF variant was 0.00040903. Only two individuals harboring the *SLC30A8* protective variant were identified in the QGP cohort. Twenty-three individuals with the APOE2-ε2ε2 genotype protective against AD were identified (Table 1). The APOE ε2 haplotype is defined by the presence of two specific single nucleotide polymorphisms (SNPs): rs429358 (T) and rs7412 (T).

**Table 1:** Allele frequency of protective variants in > 14,669 individuals from the Qatar Genome Project

Gene	Variant	rs#	AF	HOM-REF	HET	HOM-VAR
APOE	E3ch R136S	rs121918393				
	APOE2-ε2ε2 genotype	rs429358	0.0867089	12252	2281	131
		rs7412	0.0320803	13699	873	32
PCSK9	p.Tyr142*	rs67608943				
	p.Cys679*	rs28362286	0.00040903	14657	12	0
ANGPTL3	p.Glu129*	rs200785483				
	p.Ser17*	rs267606655				
APOC3	p.Arg19*	rs76353203	0.0152703	14234	422	13
SLC30A8	p.Arg138*	rs200185429	6.82E-05	14667	2	0

*AF* allele frequency, *HOM-REF* homozygous reference genotypes, *HET* heterozygous, *HOM-VAR*, count of homozygous variant genotypes

### Epigenetic aging in antigeroid syndromes

**Fig. 1:**
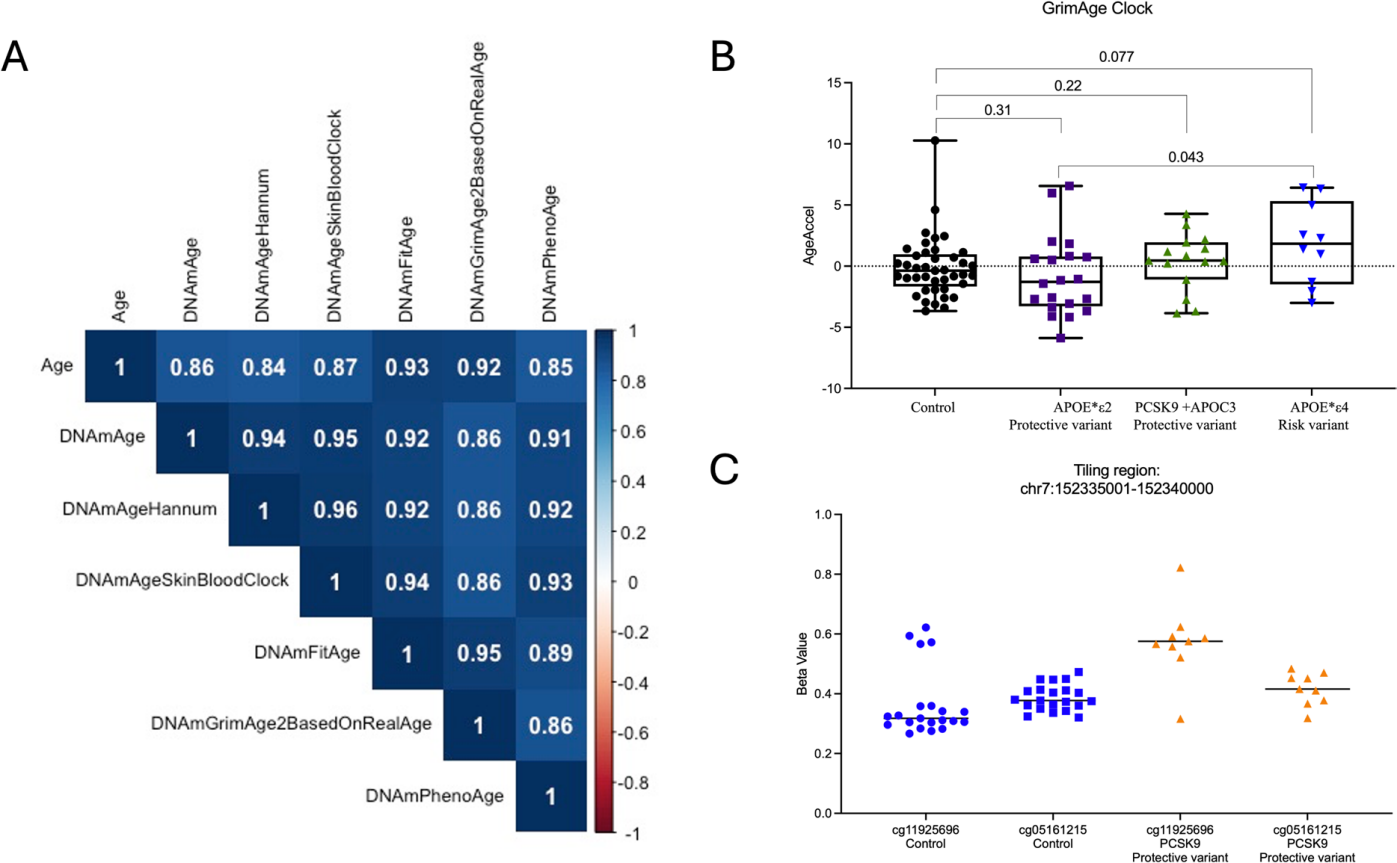
**A** Correlation coefficients (r) of estimated DNA methylation age by different DNA methylation clocks and chronological age. **B** Epigenetic age acceleration comparison between individuals carrying protective variants for atherosclerosis, protective and high-risk variants for Alzheimer’s disease, and controls using the GrimAge clock. **C** A representative of differentially methylated tiling regions between individuals with *PCSK9* protective variants vs controls

We obtained blood DNA from 6 homozygous individuals with the *APOC3* c.55C > T (p.Arg19*) variant and from 9 individuals with the *PCSK9* c.2037C > A, (p.Cys679*) variant. In addition, blood DNA was obtained from 21 individuals with the APOE ε2 haplotypes and 10 samples with the APOE ε4 allele, a variant associated with increasing risk of AD. As controls, 41 age and gender-matched control samples with the APOE ε3 allele that do not carry any other protective variants were selected. We measured DNA methylation levels using the Illumina Infinium MethylationEPIC V2 arrays in whole blood DNA of 88 healthy individuals, including the 36 that carried protective variants against AD and atherosclerosis. The DNAmAge estimates of the investigated clocks strongly correlated with chronological age (*r* = 0.84–0.93) and between the outputs of epigenetic clocks (*r* = 0.86–0.96, Fig. 1A).

Next, EAA was calculated using various epigenetic clocks to compare healthy individuals carrying the protective variants *vs* control samples. In this analysis, individuals carrying the risk APOE4 allele that leads to early AD showed a significant increase in epigenetic age measured using the GrimAge clock (*p* = 0.043) when compared to individuals harboring protective variants against AD (APOE2-ε2ε2) (Fig. 1B). Furthermore, a tendency towards significance was observed between control samples and samples carrying the AD risk allele (*p* = 0.077). Comparing the samples according to protective variant revealed no significant EAA differences between the different groups using different epigenetic clocks. Similarly, IEAA and EEAA had no significant difference between the compared groups. Next, we grouped all samples carrying protective variants and compared them to controls and to individuals carrying the AD risk allele. None of the comparisons showed a significant difference across the various studied clocks.

**Fig. 2:**
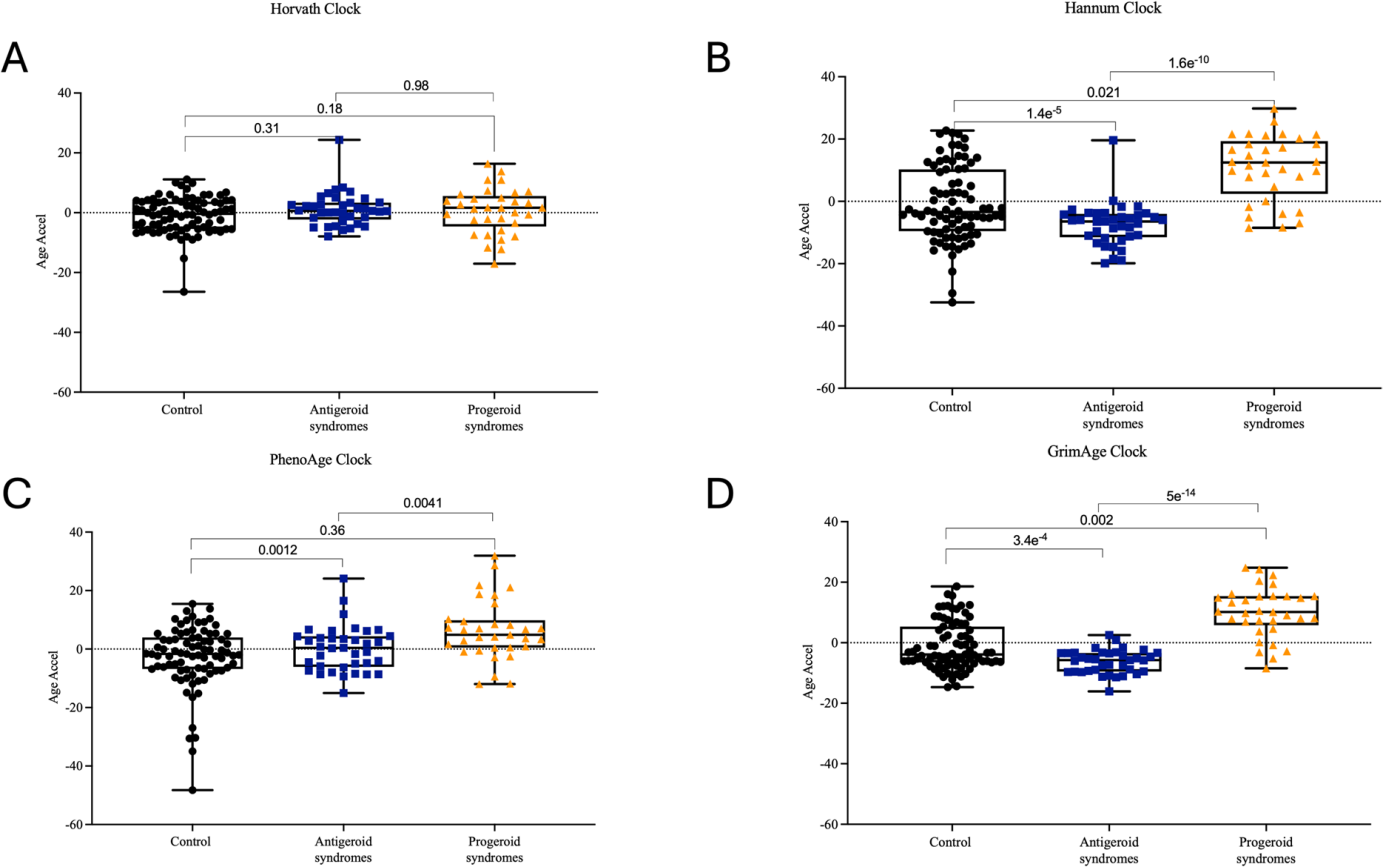
Comparing epigenetic age acceleration in antigeroid syndromes (protective variants), progeroid syndromes, and controls using the **A** Horvath, **B** Hannum, **C** PhenoAge, and **D** GrimAge clocks

We further compared EAA in samples with protective variants, i.e., antigeroid vs progeroid samples in publicly available datasets. The progeroid samples were primarily categorized into old (> 20 years) and young (≤ 20 years) to avoid age differences between groups. This analysis was conducted on progeroid samples > 20 years of age after confirming no significant age differences between this group, controls, and individuals with protective variants. The distribution of syndromes included in this analysis is presented in Supplementary Table 1. In this analysis, we observed a significant reduction in age acceleration using the Hannum (*p* = 1.6e^-10^), PhenoAge (*p* = 0.004), and GrimAge (*p* = 5e^-14 ^^−12^) clocks when comparing antigeroid vs progeroid syndromes (Fig. 2).

### Differential DNA methylation analysis in antigeroid syndrome

We performed differential DNA methylation analysis to identify differentially methylated CpG sites/regions between groups. We grouped samples according to protective variants and compared DNA methylation differences between each group vs controls. We identified 37 significant differentially methylated CpG sites with an FDR adj. *p-value* < 0.05 and β methylation difference > 0.05 (> 5% methylation difference), including 16 hypermethylated and 21 hypomethylated CpG sites (Supplementary Table 2), when comparing controls to individuals carrying the *PCSK9* protective variant. In addition, we detected two significant tiling regions with at least 2 CpG sites following FDR adjustment with > 5% methylation difference ((Fig. 1C, Supplementary Table 3). The other comparison of *APOE2-ε2ε2* and *APOC3* protective variants did not display any significant DMPs/DMRS. Gene ontology (GO) enrichment analysis for the 37 significant CpG sites revealed significant terms related to the cardiovascular system, such as heart morphogenesis (*p*-adj = 0.04) and cardiac chamber development (*p*-adj = 0.04) (Table 2). Furthermore, several GO terms related to immune response were enriched, including negative regulation of cytokine production, myeloid cell homeostasis, and cell activation involved in immune response.

**Table 2:** Gene ontology enrichment for the 37 significant differentially methylated CpGs in individuals with *PCSK9* protective variants after adjusting for the total number of CpG sites per gene

ID	Description	Size	adj. *p-value*
GO:0001818	Negative regulation of cytokine production	439	0.04
GO:0002262	Myeloid cell homeostasis	202	0.04
GO:0002263	Cell activation involved in immune response	355	0.04
GO:0002285	Lymphocyte activation involved in immune response	239	0.04
GO:0002286	T cell activation involved in immune response	130	0.04
GO:0002366	Leukocyte activation involved in immune response	349	0.04
GO:0002573	Myeloid leukocyte differentiation	266	0.04
GO:0002761	Regulation of myeloid leukocyte differentiation	135	0.04
GO:0003007	Heart morphogenesis	318	0.04
GO:0003205	Cardiac chamber development	186	0.04

*GO* gene ontology, *adj. p-value* FDR adjusted *p-value*

DNA variability analysis in the *PCSK9* protective variant group also resulted in 640 variable CpG sites, of which seven are differentially methylated and 62 variable tiling, with one being differentially methylated. Next, we performed an eForge analysis to check for overlap with DNase I hypersensitive sites in different tissues. There was a tendency for significant overlap with (*p-value* < 0.05) in psoas muscle and skin. However, the *Q*-value was non-significant (*Q*-value > 0.01) following adjustment for multiple testing. Finally, we grouped the samples according to the APOE allele and compared the 23 sample carriers of the APOE2 allele vs. 16 APOE4 allele carriers. No significant CpG sites were identified after adjusting for age, gender, and cell type composition. We checked if previously identified CpG sites between carriers of APOE4 and APOE2 alleles in a study by Walker *et al*. [27] showed nominal significance (unadjusted *p-value* < 0.05) in our analysis. Only two CpG sites, cg03793277 (*APOC1*) and cg05249393 (*LDLR*), were nominally significant in our study.

## Discussion

In this study, we conducted DNA methylation profiling using the Illumina Infinium MethylationEPIC BeadChip V2 on whole blood from 88 samples, 36 of which carried protective variants against Alzheimer’s disease and atherosclerosis selected from a large cohort of  > 14,669 samples from the Qatar Genome Project. To our knowledge, this is the first study measuring epigenetic age acceleration and DNA methylation differences in individuals with protective variants against age-related diseases. Our analysis showed a strong correlation between DNA methylation age (DNAmAge) and chronological age for first and second-generation clocks, including GrimAge and PhenoAge clocks.

The impact of the protective variants on age acceleration remains a crucial area of interest, potentially revealing whether these variants, in addition to reducing the probability of developing age-related diseases, can also systemically influence the overall biological aging process. EAA was assessed using various epigenetic clocks to compare samples harboring protective variants for diseases such as AD and atherosclerosis against control samples and those harboring the APOE4 risk allele known to predispose individuals to early-onset AD. Samples carrying risk alleles for AD exhibited a significant increase in epigenetic age as measured by the GrimAge clock compared to the group carrying protective variants, suggesting that the APOE4 risk allele is associated with accelerated biological aging, particularly in the context of AD risk. GrimAge is based on surrogate biomarkers for health-related plasma proteins, smoking pack-years, and sex or predicting mortality risk. Further analysis was conducted to compare EAA in antigeroid (carrying protective variants) vs progeroid samples. Evidently, EAA was significantly reduced in the protective variant carriers, as measured by several epigenetic clocks (Hannum, PhenoAge, and GrimAge clocks). This suggests that individuals carrying protective variants may experience slower biological aging when compared to patients with progeroid features. Some but not all progeroid syndromes have been previously shown to be associated with EAA [28, 29]. We observed significant epigenetic age differences when comparing patients with progeroid syndromes to individuals with protective variants, indicating that a single variant could have a significant impact on biological aging, particularly when studying monogenic progeroid syndromes caused by deleterious variants in a single gene.

In the subsequent differential DNA methylation analysis, we observed significant DNA methylation differences between controls and individuals carrying the *PCSK9* variant. The role of the *PCSK9* protective variant has been well established in lipid metabolism and cardiovascular protection, which aligns with the gene ontology (GO) enrichment analysis of the 37 significant CpG sites showing enrichment for heart morphogenesis and cardiac chamber development, suggesting that the protective variant may influence epigenetic regulation in pathways critical for cardiovascular development and function. These findings indicate that *PCSK9* might additionally exert specific epigenetic modifications that potentially modulates cardiovascular disease susceptibility.

The comparison between carriers of the APOE2 allele (*n* = 23) and the APOE4 allele (*n* = 16) revealed no significant differentially methylated CpG sites. To replicate findings from the Walker et al. (24) study, which reported differential methylation between APOE ε4 and APOE ε2 carriers, we checked for overlap in the identified CpG sites at the nominal *p-value* level. Only two CpG sites cg03793277 and cg05249393 were replicated in our dataset. The limited replication may be attributed to the relatively small sample size, highlighting the need for larger cohorts to detect significant methylation differences associated with APOE alleles. Interestingly, the two common significant sites are located in the *APOC1* and *LDLR* genes, which have been previously associated with increased AD risk in combination with certain APOE haplotypes in certain cases [30–34].

Although one of the limitations of our study is the low sample size, we screened more than 14,669 sequenced individuals to identify the 37 individuals harboring protective variants. Furthermore, we could only identify two individuals with T2D protective variants, of which only one sample had genomic DNA available. This prevented us from performing EAA and differential DNA methylation analysis in individuals protected against T2D. This LOF allele (p.Arg138*) in the *SLC30A8* gene increases protection against T2D by 53%. However, its frequency is very low and almost non-existent in the Qatari population, similar to the *ANGPTL3* protective variants. In addition, epigenetic age and differential DNA methylation analysis was performed in blood, therefore, we were not able to determine if other tissues (particuarly brain) exhibits similar reduced epigenetic age rates in individuals with the APOE*E2 protective variant. However, a brain specific epigenetic clock (PCBrainAge) has been previously reported to be associated with APOE4 status. Carriers of either 1 or 2 APOE ε4 alleles displayed significant epigenetic age acceleration when measured using this epigenetic clock [35]. Furthermore, standard epigenetic clocks (trained using blood DNA methylation data) do not display an effect in the dorsolateral pre-frontal cortex and are not associated with the APOE genotype [36].

In conclusion, we could observe that the APOE*E2 protective variant not only leads to AD protection but also reduces epigenetic acceleration (as measured by the GrimAge clock) compared to the AD high-risk group. This provides further evidence for a role of APOE in human longevity [37]. The ability of protective variants to decelerate biological aging and modulate epigenetic profiles offers essential insights into the interplay between genetics, epigenetics, and the aging process. Focusing on antigeroid syndromes offers an avenue to understand biological aging in those individuals with beneficial variants resistant to certain geriatric disorders. However, further research with larger sample sizes is needed to confirm these findings and explore the underlying mechanisms driving the observed changes in biological age in individuals with the protective APOE2 allele.

## Supplementary Information

Below is the link to the electronic supplementary material.

Supplementary file1 Table 1: Total number of samples according to syndrome type in the progeroid syndromes group with age > 20 years. Table 2: Differentially methylated probes in individuals harboring *PCSK9* protective variants with an FDR-adjusted *p*-value < 0.05 and β difference > 0.05. Table 3: Differentially methylated tiling regions with  2 CpG sites with FDR adj. p-value < 0.05 and methylation difference of > 5%. (DOCX 22 KB)

## Data Availability

The datasets used in the current study are available from the corresponding author upon reasonable request.
