# Supplementary material for: The role of protective genetic variants in modulating epigenetic aging
Source: GeroScience. 2025 Feb 10;47(4):5995–6004. doi: 10.1007/s11357-025-01548-2 (PMC12397041; doi:10.1007/s11357-025-01548-2)
Supplement: Supplementary file 1 — Supplementary file1 Table 1: Total number of samples according to syndrome type in the progeroid syndromes group with age > 20 years. Table 2: Differentially methylated probes in individuals harboring PCSK9 protective variants with an FDR-adjusted p-value < 0.05 and β difference > 0.05. Table 3: Differentially methylated tiling regions with 2 CpG sites with FDR adj. p-value < 0.05 and methylation difference of > 5%. (DOCX 22 KB) [file 11357_2025_1548_MOESM1_ESM.docx]

Supplementary Table 1:

| **Type** | **Number** |
| --- | --- |
| Classical Werner Syndrome | 20 |
| Atypical Werner Syndrome | 5 |
| Non-Classical HGPS | 3 |
| CTC1-related Syndrome | 2 |
| BSCL2-related Syndrome | 1 |
| Dyskeratosis Congenita | 1 |

Supplementary Table 2:

| **cgid** | **Chromosome** | **Start** | **mean.Control** | **mean.PCSK9 protective variant** | **mean.diff** | **diffmeth.p.val** | **diffmeth.p.adj** |
| --- | --- | --- | --- | --- | --- | --- | --- |
| cg00832645 | chr1 | 59215402 | 0.89 | 0.73 | 0.16 | 4.74E-09 | 0.0008 |
| cg00832652 | chr1 | 59216048 | 0.70 | 0.57 | 0.13 | 8.56E-09 | 0.0010 |
| cg01411376 | chr1 | 151512436 | 0.61 | 0.68 | -0.07 | 8.44E-07 | 0.0260 |
| cg12615916 | chr1 | 227318153 | 0.09 | 0.21 | -0.12 | 4.58E-07 | 0.0171 |
| cg10664993 | chr2 | 98192824 | 0.84 | 0.62 | 0.22 | 4.21E-07 | 0.0171 |
| cg03130559 | chr2 | 236977089 | 0.56 | 0.67 | -0.11 | 1.41E-06 | 0.0384 |
| cg23555395 | chr2 | 237127921 | 0.69 | 0.79 | -0.10 | 2.03E-06 | 0.0443 |
| cg19137817 | chr2 | 237133025 | 0.48 | 0.40 | 0.09 | 3.94E-07 | 0.0171 |
| cg08712267 | chr2 | 237141608 | 0.81 | 0.68 | 0.12 | 4.50E-09 | 0.0008 |
| cg22174197 | chr3 | 38608674 | 0.74 | 0.66 | 0.08 | 4.69E-07 | 0.0171 |
| cg10599156 | chr3 | 141426389 | 0.62 | 0.72 | -0.10 | 8.91E-07 | 0.0260 |
| cg08879826 | chr4 | 8076222 | 0.52 | 0.64 | -0.12 | 8.03E-08 | 0.0050 |
| cg20619100 | chr4 | 33104295 | 0.23 | 0.37 | -0.15 | 1.38E-08 | 0.0013 |
| cg23355053 | chr4 | 39030729 | 0.32 | 0.18 | 0.15 | 1.19E-06 | 0.0337 |
| cg08078498 | chr5 | 67248989 | 0.31 | 0.47 | -0.16 | 2.29E-06 | 0.0488 |
| cg26500480 | chr5 | 128378364 | 0.73 | 0.67 | 0.07 | 3.05E-07 | 0.0149 |
| cg06872887 | chr5 | 145835882 | 0.28 | 0.20 | 0.07 | 1.84E-06 | 0.0434 |
| cg09546802 | chr5 | 145835983 | 0.38 | 0.25 | 0.13 | 1.61E-06 | 0.0421 |
| cg11609571 | chr5 | 145836066 | 0.49 | 0.33 | 0.16 | 2.39E-07 | 0.0123 |
| cg11840968 | chr5 | 176740282 | 0.84 | 0.90 | -0.06 | 5.51E-09 | 0.0008 |
| cg01203153 | chr5 | 181216432 | 0.18 | 0.09 | 0.09 | 2.85E-08 | 0.0019 |
| cg22208713 | chr7 | 44154867 | 0.48 | 0.28 | 0.21 | 2.39E-06 | 0.0498 |
| cg14244967 | chr7 | 99450603 | 0.85 | 0.73 | 0.12 | 1.73E-06 | 0.0421 |
| cg11716000 | chr7 | 131541093 | 0.75 | 0.50 | 0.26 | 3.23E-10 | 0.0001 |
| cg11924147 | chr7 | 152150860 | 0.84 | 0.48 | 0.36 | 1.33E-10 | 0.0001 |
| cg11925696 | chr7 | 152335946 | 0.32 | 0.57 | -0.26 | 1.46E-12 | 0.0000 |
| cg08430157 | chr9 | 38142078 | 0.78 | 0.70 | 0.08 | 3.40E-07 | 0.0156 |
| cg02279396 | chr9 | 137071043 | 0.32 | 0.42 | -0.10 | 8.74E-09 | 0.0010 |
| cg12389611 | chr11 | 46330356 | 0.66 | 0.53 | 0.13 | 1.42E-07 | 0.0078 |
| cg16402382 | chr11 | 61446460 | 0.32 | 0.45 | -0.13 | 8.77E-07 | 0.0260 |
| cg13456836 | chr13 | 23932660 | 0.48 | 0.63 | -0.15 | 1.74E-08 | 0.0013 |
| cg26850807 | chr16 | 88453143 | 0.10 | 0.15 | -0.06 | 1.57E-08 | 0.0013 |
| cg22052140 | chr17 | 2900222 | 0.66 | 0.51 | 0.14 | 1.93E-06 | 0.0443 |
| cg02885454 | chr18 | 13513360 | 0.50 | 0.60 | -0.10 | 5.65E-07 | 0.0190 |
| cg01213435 | chr18 | 62457968 | 0.74 | 0.58 | 0.16 | 5.08E-07 | 0.0178 |
| cg06095304 | chr18 | 74064693 | 0.48 | 0.55 | -0.07 | 1.73E-06 | 0.0421 |
| cg13323440 | chr22 | 20066608 | 0.82 | 0.65 | 0.17 | 1.64E-06 | 0.0421 |

Supplementary Table 3:

| Chromosome | Start | End | mean Control | Mean PCSK9 | mean difference | comb.p.val | comb.p.adj.fdr | num.sites |
| --- | --- | --- | --- | --- | --- | --- | --- | --- |
| chr1 | 59215001 | 59220000 | 0.80 | 0.71 | 0.09 | 3.25E-08 | 0.0028 | 3 |
| chr7 | 152335001 | 152340000 | 0.35 | 0.50 | -0.15 | 1.64E-09 | 0.0004 | 2 |
